# Post-inhaled corticosteroid pulmonary tuberculosis and pneumonia increases lung cancer in patients with COPD

**DOI:** 10.1186/s12885-016-2838-4

**Published:** 2016-10-10

**Authors:** Ming-Fang Wu, Zhi-Hong Jian, Jing-Yang Huang, Cheng-Feng Jan, Oswald Ndi Nfor, Kai-Ming Jhang, Wen-Yuan Ku, Chien-Chang Ho, Chia-Chi Lung, Hui-Hsien Pan, Min-Chen Wu, Yung-Po Liaw

**Affiliations:** 1 School of Medicine, Chung Shan Medical University, Taichung City, Taiwan; 2 Divisions of Medical Oncology and Pulmonary Medicine, Chung Shan Medical University Hospital, Taichung City, Taiwan; 3 Department of Public Health and Institute of Public Health, Chung Shan Medical University, Taichung City, Taiwan; 4 Office of Physical Education, Chung Yuan Christian University, Taoyuan City, Taiwan; 5 Department of Neurology, Lu-Tung Christian Hospital, Changhua, Taiwan; 6 Department of Physical Education, Fu Jen Catholic University, New Taipei City, Taiwan; 7 Department of Family and Community Medicine, Chung Shan Medical University Hospital, No. 110, Sec. 1 Jianguo N. Rd., Taichung City, 40201 Taiwan; 8 Department of Pediatrics, Chung Shan Medical University Hospital, Taichung City, Taiwan

**Keywords:** Chronic obstructive pulmonary disease, Inhaled corticosteroid, Pneumonia, Tuberculosis

## Abstract

**Background:**

Inhaled corticosteroids (ICS) have been associated with decreased lung cancer risk. However, they have been associated with pulmonary infections (tuberculosis [TB] and pneumonia) in patients with chronic obstructive pulmonary disease (COPD). TB and pneumonia have increased lung cancer risk. The association between post-ICS pulmonary infections and lung cancer remains unclear.

**Methods:**

We conducted a retrospective cohort study from 2003 to 2010 using the Taiwan National Health Insurance Research Database. Among the 1,089,955 patients with COPD, we identified 8813 new users of ICS prescribed for a period of 3 months or more and 35,252 non-ICS users who were randomly matched for sex, age and date of ICS use from 2003 to 2005. Cox proportional hazard regression was used to estimate the hazard ratio (HR) of pulmonary infections in patients with/without ICS use.

**Results:**

The HRs for lung cancer in ICS users with sequential lung infections were as follows; 2.42 (95 % confidence interval [CI], 1.28–4.58) for individuals with TB, 2.37 (95 % CI, 1.01–5.54) for TB and pneumonia, and 1.17(95 % CI, 0.69–1.98) for those with pneumonia. For non-ICS users with pulmonary infections, the HRs were 1.68 (95 % CI, 0.78–3.65) for individual with TB and pneumonia, 1.42 (95 % CI, 0.89–2.26) for TB, and 0.95 (95 % CI, 0.62–1.46) for individuals with pneumonia.

**Conclusions:**

COPD patients with TB /or pneumonia who used ICS had increased risk of lung cancer. Because the overall prognosis of lung cancer remains poor, screening tests are recommended for patients with these conditions.

## Background

The prevalence of chronic obstructive pulmonary disease (COPD) in Taiwan is 2.48 % [1]. COPD is a common chronic inflammatory airway disease and is associated with lung cancer [2, 3]. Inhaled (ICS) and oral corticosteroids (OCS) have been used to reduced airway inflammation and acute exacerbations [4–6]. Lee et al. conducted a nested case-control study with new adult users of ICS. Results showed that ICS use led to a reduced risk of lung cancer [7]. In a separate study, ICS has also reduced lung cancer risk among COPD patients [8] and those who quit smoking [9].

However, there is a close association between ICS use, pulmonary tuberculosis (TB) [10] and pneumonia [11]. TB [12] and pneumonia [13] have been associated with increased risk of lung cancer. Coexistence of COPD and TB correlates with increased incidence and mortality of lung cancer [2, 14]. The association between post-ICS pulmonary infections and lung cancer in patients with COPD remains unclear. In this study, we evaluated the association between post-ICS pulmonary infections and lung cancer using the National Health Insurance Research Database (NHIRD).

## Methods

### Data source

Data from the NHIRD, Taiwan Cancer Registry Database (TCRD) and the National Death Registry Database (NDRD) were linked in this retrospective cohort study. The NHIRD provide a comprehensive health care information including diagnoses, clinical visits, admission and prescriptions. The multiple databases were used to assess the age at cancer onset, person-month follow-up, death, survival time, and misdiagnosis. Personal information including ethnicity, family history, lifestyle, occupation, and habits such as smoking and alcohol intake was not available in the NHIRD.

### Ethics

This study was approved by the Institutional Review Board of the Chung-Shan Medical University Hospital. The informed consent was waived by the Institutional Review Board as the source data were encrypted and the data extracted were anonymous.

### COPD patients with ICS use

This study enrolled patients with COPD from 2001 to 2005 who were free from lung cancer before 2002. Excluded were COPD patients who used ICS before 2002 and those with incomplete information. Also exclude were patients below 20 and over 100 years of age. We identified patients who were prescribed ICS and OCS from 2003 to 2005 using the inpatient and outpatient medical records. Information regarding ICS and OCS prescription were also collected, including prescription dates, daily dose prescribed and the duration of prescription. The ICS included beclomethasone, budesonide, fluticasone and ciclesonide whether used alone or in a combination inhaler with an inhaled β2 agonist. Eligible participants included COPD patients who were first time users of ICS prescribed for a period of 3 months or more. The date of the first use of ICS was called the index date.

For each new ICS user, four controls were randomly matched for sex, age and index date without replication from COPD patients who were not exposed to ICS. The eligible participants (ICS and non-ICS users) were followed up until the development of lung cancer, loss to follow-up, death, or the end of the year 2010.

### Post- ICS pulmonary TB and pneumonia

Pulmonary TB was defined by a compatible International Classification of Diseases, Ninth Revision, Clinical Modification (ICD-9-CM) code (010-012, 018, and137) with either two outpatient visits or one admission after the index date. Cases of pneumonia were using the ICD-9-CM codes: 480–486, and 487.0. Patients diagnosed with TB or pneumonia before or within 3 months after the index date were also excluded.

### Outcomes

The primary outcome was the first diagnosis of lung cancer during the follow-up. Lung cancer was defined by a compatible ICD-9-CM code 162. The cell types of lung cancer were further identified using the TCRD. Further exclusions included patients who either died or had lung cancer within 2 years of the index date.

### Medications

To define OCS use, patients who took a cumulative dose of 1680 mg (or 60 mg daily for 4 weeks) of hydrocortisone equivalents or more during 1 year after the index date were enrolled [10]. All OCS received during follow-up were converted to the equivalent dose of hydrocortisone in milligrams (4 mg of hydrocortisone = 5 mg of cortisone = 1 mg of prednisolone = 0.8 mg methylprednisolone = 0.8 mg of triamcinolone = 0.4 mg of paramethasone = 0.15 mg of betamethasone = 0.15 mg of dexamethasone) [15].

In addition, we also adjusted for the severity of COPD medications, including short-acting inhaled β2 agonists (SABAs; salbutamol, fenoterol, procaterol, or terbutaline), long-acting inhaled β2 agonists (LABAs; salmeterol, formoterol, indacaterol, or olodaterol), and theophylline.

### Variables of exposure

Comorbidities were defined by either two outpatient visits or one hospitalization in 1 year. They included COPD (ICD-9-CM: 490, 491, 492, 494, and 496), chronic kidney disease (ICD-9-CM: 585 and 586), diabetes mellitus (ICD-9-CM: 250), hyperlipidemia (ICD-9-CM: 272), liver cirrhosis (ICD-9-CM: 571.2, 571.5, and 571.6), smoking-related cancers (ICD-9-CM: 140–150, 157, 160–161, and 189), autoimmune disease (ICD-9-CM: 710 and 714), atopic dermatitis (ICD-9-CM: 691), and rhinosinusitis (ICD-9-CM codes: 472.0, 473, and 477). In order to assess the severity of COPD, the number of outpatient and inpatients visits for respiratory diseases during 2 years after the index date were evaluated. However, information regarding lifestyle behavior such as smoking was not available in the NHIRD, hence preventing direct adjustment for possible confounders.

### Statistical analysis

Data analysis was made using the SAS 9.3 software (SAS Institute, Cary, NC). Differences in baseline characteristics and comorbidities between ICS and non-ICS users were compared using the Chi-square test and *t*-test. Kaplan-Meier survival plots were used to evaluate the effect of predictor variables on lung cancer at the univariate level. ICS users and non-users were compared using the log-rank test. The adjusted hazard ratios (HRs) and 95 % confidence intervals (CIs) of the lung cancer risk factors were calculated using multivariate Cox proportional hazards regression modeling. A *P*-value of less than 0.05 was considered to be statistically significant.

## Results

**Fig. 1:**
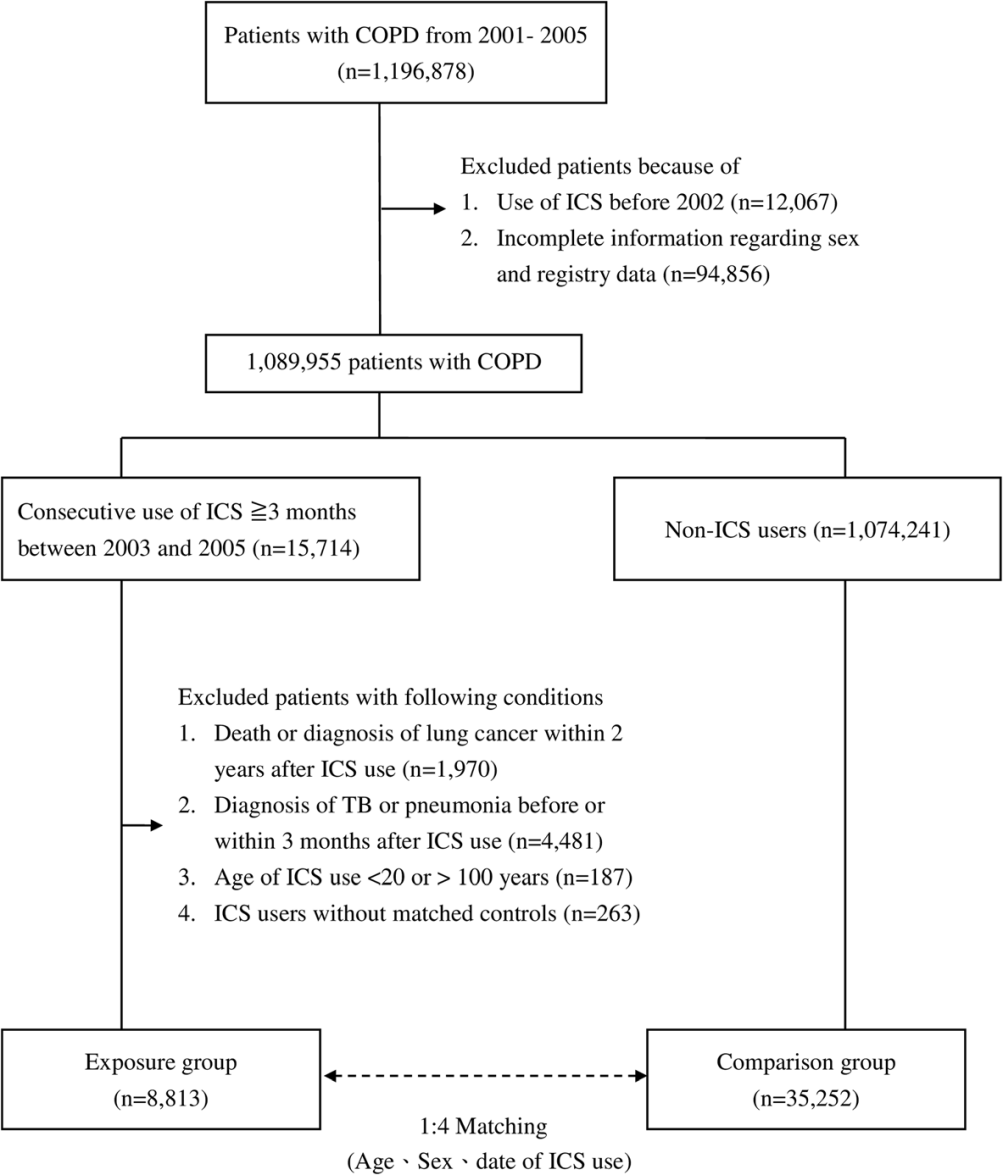
Flow diagram of the enrollment process. COPD, chronic obstructive pulmonary disease; ICS, inhaled corticosteroid, TB, tuberculosis

We identified 1,196,878 patients with COPD from 2001 to 2005 who were not diagnosed with lung cancer before 2002. We excluded 12,067 patients who received ICS before 2002 and 94,856 patients with incomplete information including sex and registry data. We enrolled 15,714 COPD patients who used ICS for a period of 3 months or more. Further exclusion included the following: participants who died or those that were diagnosed with lung cancer within 2 year after the index date (*n* = 1970), individuals diagnosed with TB or pneumonia before or 3 months after the index date (*n* = 4481), people below 20 and above 100 years of age (*n* = 187), and ICS users without matched controls (*n* = 263). Therefore, 8813 new users of ICS were matched with 35,252 non-users (Fig. 1).

Information on demographic characteristics, medications, comorbidities, and follow-up durations of the study participants are shown in Table 1. In total, 179 ICS users (i.e. no lung infection, 143 cases; pneumonia, 19; TB, 11, and TB + pneumonia, 6) and 496 non-users had lung cancer (no lung infection, 442 cases; pneumonia, 28; TB, 19, and TB + pneumonia, 7).

**Table 1:** Characteristics of the Study Population

	ICS (*N* = 8813)	No ICS (*N* = 35,252)	*P*-value
Pulmonary infection combinations (%)			<0.001
None	7823 (88.7)	32,694 (92.7)	
Pneumonia	703 (8.0)	1654 (4.7)	
TB	182 (2.1)	678 (1.9)	
TB + pneumonia	105 (1.2)	226 (0.7)	
Medications (%)			
OCS	3376 (38.3)	1992 (5.7)	<0.001
LABA	4236 (48.1)	1186 (3.4)	<0.001
SABA	7313 (83.0)	6244 (17.7)	<0.001
Theophylline	8305 (94.2)	19,876 (56.4)	<0.001
Statins	1368 (15.5)	5591 (15.7)	0.437
Aspirin	2652 (30.1)	12,203 (34.6)	<0.001
Sex (%)			1.000
Men	6078 (69.0)	24,312 (69.0)	
Women	2735 (31.0)	10,940 (31.0)	
Age (years, %)			1.000
20–39	270 (3.1)	1080 (3.1)	
40–59	1875 (21.3)	7500 (21.3)	
60–79	5328 (60.4)	21,312 (60.4)	
≧80	1340 (15.2)	5360 (15.2)	
Comorbidities (%)			
Diabetes	254 (2.9)	1192 (3.4)	0.019
Hyperlipidemia	1627 (18.5)	7423 (21.1)	<0.001
Chronic kidney disease	1472 (16.7)	7028 (19.9)	<0.001
Smoking-related cancers	124 (1.4)	656 (1.9)	0.004
Liver cirrhosis	96 (1.1)	423 (1.2)	0.389
Autoimmune disease	280 (3.2)	1002 (2.8)	0.095
Atopic dermatitis	162 (1.8)	734 (2.1)	0.147
Rhinosinusitis	3955 (44.9)	6739 (19.1)	<0.001
No. of outpatient visits for respiratory diseases within 2 years after index date (%)^a^	24.5 ± 14.5	6.0 ± 9.6	<0.001
≤ 15	2054 (23.3)	30,735 (87.2)	<0.001
> 15	6759 (76.7)	4517 (12.8)	
No. of inpatient visits for respiratory diseases within 2 years after index date (%)^a^	0.8 ± 1.7	0.2 ± 0.8	<0.001
0	5779 (65.6)	30,661 (87.0)	<0.001
≥ 1	3034 (34.4)	4591 (13.0)	
Urbanization (%)			<0.001
High	4934 (56.0)	18,971 (53.8)	
Mid	2830 (32.1)	11,441 (32.5)	
Low	1049 (11.9)	4840 (13.7)	
Death in 2004–2008 (%)	1118 (12.7)	3590 (10.2)	<0.001
Follow-up time (person-months)	4.2 × 10^5^	17.4 × 10^5^	
No. of lung cancer	179	496	
Incidence rate (per 10^5^ person months) (95 % C.I.)	42.2 (36.5–48.9)	28.5 (26.1–31.1)	<0.001
Histologic type (%)			0.148
Squamous cell carcinoma	34 (19.0)	128 (25.8)	
Adenocarcinoma	76 (42.5)	173 (34.9)	
Small cell carcinoma	24 (13.4)	57 (11.5)	
Others	45 (25.1)	138 (27.8)	

*CI* confidence interval, *HR* hazard ratio, *ICS* inhaled corticosteroid, *LABA* long-acting inhaled beta-agonist, *OCS* oral corticosteroid, *SABA* short-acting beta-agonist, *TB* pulmonary tuberculosis

^a^Index date was defined as the date of initiation of ICS

**Fig. 2:**
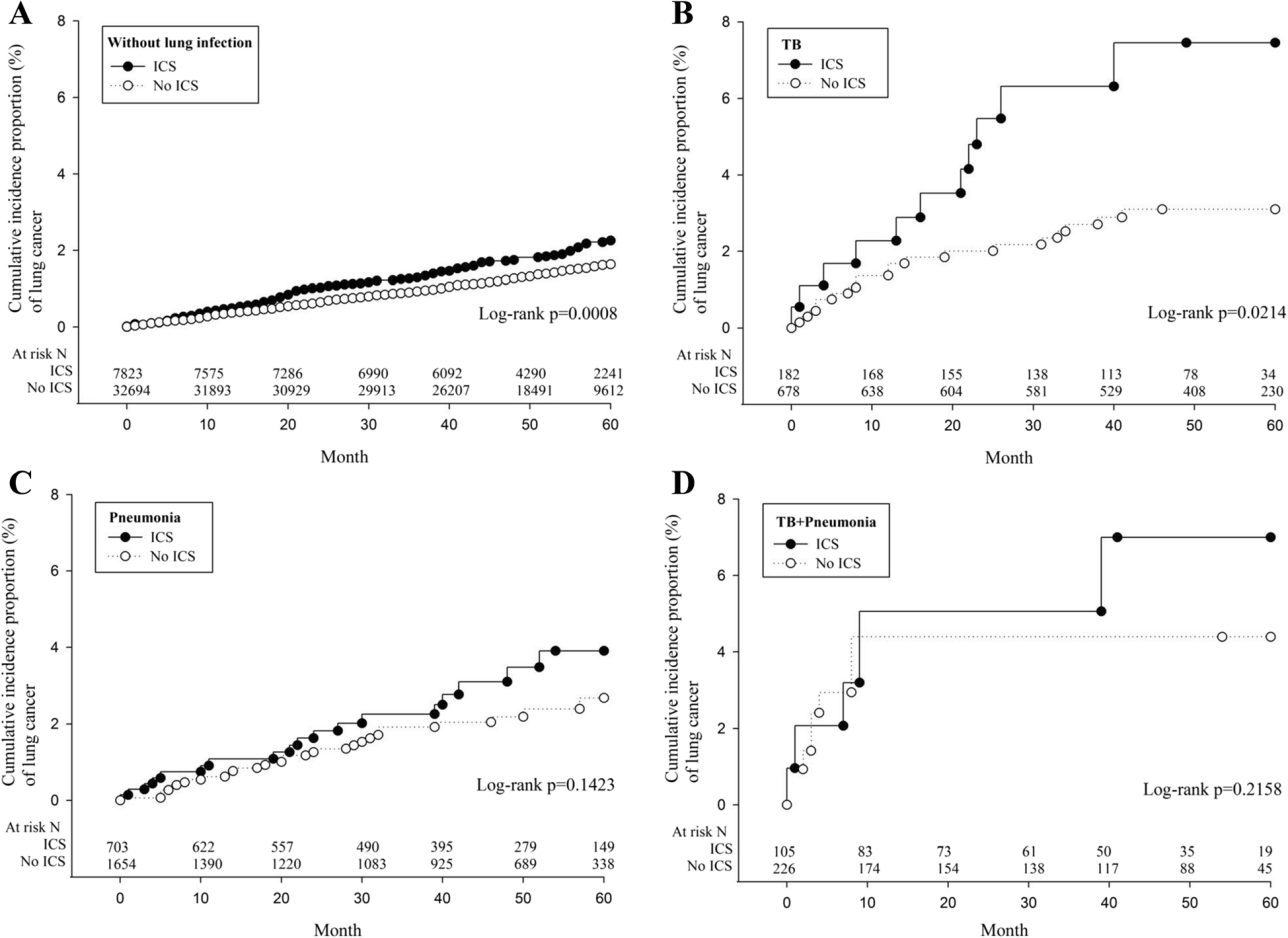
Cumulative incidence of lung cancer in ICS users and the comparison cohorts stratified by the presence of non-lung infection (**a**), TB (**b**), pneumonia (**c**), and TB+ pneumonia (**d**). ICS, inhaled corticosteroid; TB, tuberculosis

Kaplan-Meier plots for TB, pneumonia, and TB + pneumonia stratified by ICS use are presented in Fig. 2. The 5-year cumulative incidence of lung cancers was significantly higher in ICS users without lung infection than their non-user counterparts (2.3 versus 1.6 %; *p* = 0.0008), and in users with TB than their non-user counterparts (7.5 versus 3.1 %; *p* = 0.0214).

Table 2 shows the adjusted HRs of lung cancer in COPD patients with TB, pneumonia and TB + pneumonia who were users and non-users of ICS. Post-ICS TB and TB + pneumonia significantly increased the risk of lung cancer. The respective HRs were 2.42 (95 % CI, 1.28–4.58) and 2.37 (95 % CI, 1.01–5.54). There was no significant increase in lung cancer risk among ICS users without lung infection (HR, 0.88; 95 % CI, 0.67–1.14). There was no significant association between lung cancer and pulmonary infections such as TB (HR, 1.42; 95 % CI, 0.89–2.26), pneumonia (HR, 0.95; 95 % CI, 0.62–1.46) and TB + pneumonia (HR, 1.68; 95 % CI, 0.78–3.65) among the non-ICS users. No significant interaction was found between ICS use, TB (*p* = 0.084), pneumonia (*p* = 0.259), and TB + pneumonia (*p* = 0.386) (Table 3).

**Table 2:** Hazard Ratio and 95 % Confidence Intervals of Lung Cancer According to ICS and Pulmonary Infection Combinations in Patients with COPD

	All patients with COPD	
	HR (95 % CI)	*P*-value
Pulmonary infection combinations		
None	Reference	
Only ICS	0.88 (0.67–1.14)	0.314
Only pneumonia	0.95 (0.62–1.46)	0.818
Only TB	1.42 (0.89–2.26)	0.145
TB+pneumonia	1.68 (0.78–3.65)	0.187
Post-ICS pneumonia	1.17 (0.69–1.98)	0.554
Post-ICS TB	2.42 (1.28–4.58)	0.007
Post-ICS TB+pneumonia	2.37 (1.01–5.54)	0.046
Medications		
OCS	0.91 (0.72–1.16)	0.442
LABA	1.18 (0.93–1.50)	0.177
SABA	1.32 (1.07–1.64)	0.010
Theophylline	1.21 (0.99–1.48)	0.052
Statins	1.05 (0.82–1.35)	0.704
Aspirin	0.89 (0.76–1.05)	0.173
Sex		
Men	2.76 (2.20–3.48)	<0.001
Women	Reference	
Age group		
20–39	-	-
40–59	Reference	
60–79	4.69 (3.28–6.69)	<0.001
≧80	6.47 (4.42–9.48)	<0.001
Urbanization		
High	Reference	
Mid	1.05 (0.89–1.24)	0.575
Low	0.97 (0.77–1.23)	0.799
Comorbidities		
Chronic kidney disease	1.35 (0.94–1.93)	0.102
Diabetes	0.99 (0.82–1.21)	0.967
Hyperlipidemia	1.08 (0.85–1.36)	0.544
Liver cirrhosis	0.84 (0.43–1.62)	0.599
Smoking-related cancers	1.57 (0.91–2.73)	0.107
Autoimmune disease	1.57 (1.07–2.32)	0.022
Atopy dermatitis	0.67 (0.36–1.25)	0.208
Rhinosinusitis	0.90 (0.75–1.08)	0.263
No. of outpatient visits for respiratory diseases within 2 years after index date^a^		
≤15	Reference	
>15	1.27 (1.03–1.56)	0.025
No. of inpatient visits for respiratory diseases within 2 years after index date^a^		
0	Reference	
≥1	1.07 (0.85–1.35)	0.582

Reference was defined as the reference group

*CI* confidence interval, *COPD* chronic obstructive pulmonary disease, *HR* hazard ratio, *ICS* inhaled corticosteroid, *LABA* long-acting inhaled beta-agonist, *OCS* oral corticosteroid, *SABA* short-acting beta-agonist, *TB* pulmonary tuberculosis

^a^Index date was defined as the date of initiation of ICS

**Table 3:** Interaction between ICS use and lung infections

	HR (95 % CI)		p for ICS x pulmonary infection interaction
	No ICS	ICS	
Model 1			
No lung infection	1	0.89 (0.68–1.16)	0.084
TB	1.42 (0.89–2.26)	2.48 (1.31–4.72)	
Model 2			
No lung infection	1	0.88 (0.67–1.15)	0.259
Pneumonia	0.93 (0.60–1.44)	1.17 (0.69–1.99)	
Model 3			
No lung infection	1	0.88 (0.67–1.16)	0.386
TB+pneumonia	1.65 (0.76–3.58)	2.38 (1.01–5.58)	

Each model was adjusted by sex, age, medications, comorbidities, inpatient and outpatient visits for respiratory diseases, and urbanization

## Discussion

Corticosteroids are used to control airway inflammation in patients with COPD. They have also increased the risk of pulmonary TB and pneumonia [10, 11]. Many studies have documented a possible link between chronic inflammation, infection and lung cancer [2, 16, 17]. However, little is known about post-ICS pulmonary infections and lung cancer. Results from this study suggest that post-ICS TB with/without pneumonia may serve as risk factors for lung cancer.

COPD, a chronic disease characterized by a chronic inflammation of the lower airways has been associated with lung cancer [18]. The presence of moderate-to-severe obstructive pulmonary function was associated with a higher risk of lung cancer (HR, 2.8; 95 % CI, 1.8–4.4) [19]. Denholm et al. pooled information from seven case–control studies with 12,739 case subjects and 14,945 controls and found that chronic bronchitis and emphysema were positively associated lung cancer in men at odds ratios (ORs) of 1.33 (95 % CI, 1.20–1.48) and 1.50 (95 % CI, 1.21–1.87), respectively [20]. In a study comprising 15,219,024 Taiwanese residents, an increased risk of lung cancer was found in men (HR, 1.56; 95 % CI, 1.51–1.61) and women (HR, 1.33; 95 % CI, 1.26–1.10) with COPD [3].

ICS has been established in the treatment of COPD especially in symptomatic patients who experience useful gains in the quality of life, reduction in acute exacerbations, and an attenuation of the yearly rate of deterioration in lung function [21]. Acute severe exacerbations require the addition of systemic corticosteroids to control respiratory symptoms and improve lung function [22]. In an analysis including new adult ICS users (9177 cases and 37,048 controls), ICS use had a significant linear association with a decreased lung cancer incidence (OR, 0.79; 95 % CI, 0.69–0.90) [7]. In an epidemiologic study involving 10,474 veterans with COPD, a dose-response relationship was observed between ICS exposure and lung cancer [8]. Participants (*n* = 219) who received high-dose ICS (triamcinolone ≧1200 ug/day) had a decrease risk of lung cancer (HR, 0.39; 95 % CI, 0.16–0.96). After excluding subjects who had a lung cancer diagnosis within 1 year after enrollment, there was no significant dose-response reduction in lung cancer risk even at the higher doses of ICS. A retrospective cohort study of patients with a first-time diagnosis of COPD who had quit smoking and were regular users of ICS found a risk reduction when assessing a dose-response relationship of lung cancer [9]. The HRs were 0.88 (95 % CI, 0.51–1.52) and 0.51 (95 % CI, 0.30–0.84) in ICS users with 1–2 and 3 or more prescriptions/year, respectively. In our study, there was no decreased risk of lung cancer in ICS users with no pulmonary infections.

Immunosuppressive effects of glucocorticoids include inhibition of macrophage differentiation, production of cytokines, tumoricidal and microbicidal activities of activated macrophages, and T-cell activation [23]. The joint statement of the American Thoracic Society and the Centers for Disease Control and Prevention acknowledges that an administration of prednisone ≧ 15 mg/day (or its equivalent of another steroids) for a period of 1 month or more serves as a risk factor for TB [24]. A retrospective cohort study reported that ICS use was an independent risk factor for the development of pulmonary TB in patients who had normal chest radiographs (HR, 9.08; 95 % CI, 1.01–81.43) and in those who had radiologic findings of previous pulmonary TB (HR, 24.95; 95 % CI, 3.09–201.37) [25]. In a nested case-control study with 4139 TB cases and 20,583 controls, ICS use had a significant linear association with increased risk of TB: The OR was 1.20 (95 % CI, 1.08–1.34) [10]. In Taiwan, Chung et al. reported that there was a multiplicatively increased risk of TB in patients who used ICS and OCS compared to their non-user counterparts (OR, 4.31; 95 % CI, 3.39–5.49) [26]. Moreover, ICS has been associated with pneumonia in patients with COPD. In a nested case-control study of patients≧65 years, current users of ICS were 1.38 (OR, 1.38; 95 % CI, 1.31–1.45) times more likely to have a hospitalization of pneumonia [11]. A study has reported a gradual decrease in acute exacerbation rate of COPD and an increased incidence of pneumonia after ICS use (i.e. from 0.10 to 0.21 event/person-year) [27].

Patients with newly diagnosed TB were at increased risk of lung cancer with an adjusted HR of 3.32 (95 % CI, 2.70–4.09) [28]. Nonsmokers with TB had a significant association with lung squamous cell carcinoma and adenocarcinoma for both genders, whereas male smokers with TB were associated with squamous cell carcinoma, small cell carcinoma, and adenocarcinoma, and female smokers with TB were associated with adenocarcinoma [29]. In a separate study that recruited patients with pneumonia (22,034 patients and 88,136 matched controls), pneumonia was associated with an increased risk of lung cancer (HR, 4.24; 95 % CI, 3.96–4.55) [13]. In a meta-analysis, the relative risks of lung cancer in patients with a previous history of pneumonia and TB were 1.43 (95 % CI, 1.22–1.68) and 1.76 (95 % CI, 1.49–2.08), respectively [30]. When the analysis was restricted to nonsmokers, effects remained significant for pneumonia 1.36 (95 % CI, 1.10–1.69) and TB 1.90 (95 % CI, 1.45–2.50). In this study, increased risks of lung cancer were observed mainly in patients with post-ICS TB and TB + pneumonia.

Jian et al. reported a stronger association between coexisting COPD and TB and lung cancer [2]. The HRs were 2.42 (95 % CI, 2.18–2.69) in men and 2.41 (95 % CI,1.90–3.07) in women. Biologically, the additive effects of ICS, COPD, pneumonia, and TB on lung cancer may be explained by corticosteroids-induced compromised immune clearance of *Mycobacterium tuberculosis,* bacteria and malignant cells, and COPD and TB-related chronic inflammatory processes of the lung. More studies ought to be conducted to investigate the association between post-ICS pulmonary infections and lung cancer.

This study had several strengths. First, the sample size was large and the period of follow-up was long, hence reducing the likelihood of selection biases. Second, lung cancer was confirmed histologically, hence allowing the little possibility of misclassification. Third, with at least 2 years from the initiation of ICS and diagnosis of lung cancer, the chances of misclassifications were less.

Nevertheless, there were certain limitations. First, information on medication were assessed solely by refills, not by whether the subjects actually used the medication that were prescribed. Second, information regarding laboratory and image findings including airflow obstruction by spirometry and chest X-ray findings for TB were not available in the NHIRD. Third, frequent hospital visits might have led to a higher detection rate of TB and early-stage lung cancer. Fourth, the databases do not contain detailed information regarding smoking history, radon exposure, occupational exposures, diet preference, and family history, all of which may be risk factors for lung cancer. When looking at COPD and lung cancer risk, pack-years of cigarette smoking is critical.

## Conclusions

We found that post-ICS TB with/without pneumonia conferred a higher risk of lung cancer in COPD patients. Because of the high mortality of lung cancer, cancer screening is recommended for COPD patients with post-ICS TB with/without pneumonia.

## Abbreviation

CI: Confidence interval
COPD: Chronic obstructive pulmonary disease
HR: Hazard ratio
ICD-9-CM: International classification of diseases, ninth revision, clinical modification code
ICS: Inhaled corticosteroid
LABA: Long-acting inhaled β2 agonists
NDRD: National death registry database
NHIRD: National Health Insurance Research Database
OCS: Oral corticosteroid
OR: Odds ratio
RR: Relative risk
SABA: Short-acting inhaled β2 agonists
TB: Tuberculosis
TCRD: Taiwan cancer registry database
